# Implementation Barriers to Effective Nursing Interventions in Oncology Nursing Care in Saudi Arabia: A CMO Realist Evaluation

**DOI:** 10.3390/healthcare13212688

**Published:** 2025-10-23

**Authors:** Fatmah Jabr Alsolami

**Affiliations:** Faculty of Nursing, Umm Al-Qura University, Makkah 24382, Saudi Arabia; fjsolami@uqu.edu.sa

**Keywords:** acute care, barriers, nursing interventions, patient outcomes

## Abstract

**Background:** Nursing interventions are important in improving patient outcomes, especially in acute care units where patients encounter severe and complicated health problems. However, multiple barriers can hinder the accurate assessment of the effectiveness of such interventions. **Aim:** The aim of this study was to explore the barriers to evaluating the impact of nursing interventions on patient outcomes in acute care settings. **Methods:** This study employed a qualitative exploratory research design. This study was carried out in the acute care departments of a governmental tertiary hospital in the Western Region, Saudi Arabia. A purposive sample of 20 nurses was considered. Data were collected using a semi-structured interview guide. Thematic analysis was employed for data analysis. **Results:** The thematic analysis results identified five major themes: a lack of a standardised evaluation tool, time constraints, resource limitations, patient variability, and a lack of interdisciplinary collaboration. **Conclusions:** The results reveal that there are obstacles to evaluating nursing interventions in acute care. Such obstacles hinder the introduction of evidence-based changes in nursing practice and, consequently, affect the quality of care provided to patients. Healthcare settings should therefore focus on addressing the identified barriers and enabling nurses to effectively evaluate their care interventions.

## 1. Introduction

Nursing interventions form the basis of achieving better patient outcomes, especially in an oncology nursing setting, where the patients admitted usually have complex life-threatening conditions [1]. In emergency departments, intensive care units (ICUs), and surgery wards, referred to as acute care settings [2], rapid high-quality interventions are needed to ensure the management of critical health conditions [3]. The role of nurses primarily includes delivering direct patient care, administering treatments, giving emotional support, coordinating care, and supporting interdisciplinary teams. Nursing interventions are important, and the effects of direct patient outcomes are highly valued in acute care settings [4].

Determining the effectiveness of nursing interventions is key to developing clinical practice, patient safety, and quality care [5]. The assessment of nursing practices gives useful information on how responsible such practices are, contributes to the error-free implementation of care processes, and promotes the creation of evidence-based guidelines [6,7]. In addition, effective assessments of nursing interventions will allow healthcare organisations to evaluate the quality of healthcare provided, determine its weak points, and employ resources more effectively [1]. However, numerous obstacles exist to the assessment of nursing interventions that hinder efforts to determine their real effects on patient outcomes [8] (Hamad et al., 2025).

Although the efficacy of healthcare interventions has been addressed in several studies [2,4,7], a significant gap remains in terms of addressing the issues that nurses encounter when assessing their interventions in acute care. The existing empirical evidence highlights clinical outcomes, such as mortality, length of stay, and readmission rates, and primarily reports the interventions made by nurses in realising these outcomes [9,10]. However, the evidence to date does not account for the barriers faced by nurses in evaluating the effectiveness of such interventions. Moreover, most research has focused on chronic care management [11], resulting in the limited exploration of the effectiveness of nursing interventions in oncology clinical settings.

Some studies have already considered the general issues that surround the evaluation of healthcare interventions [12]; however, few scholars have considered the specifics of the oncology care setting, where the rates of patient turnover and pressure on nurses are high [13,14]. Furthermore, the body of research does not sufficiently address the opinions of nurses in acute care facilities. Therefore, little is known about the real barriers to evaluating the effects of nursing interventions. Thus, the major gap in the literature is the limited evidence concerning barriers to evaluating the effectiveness of nursing interventions in oncology clinical practice, especially as perceived by the nurses who are actively concerned with patient care. An awareness of these obstacles is important in creating effective responses to enhance the assessment process and ensure that nursing interventions can be effectively evaluated.

Despite the availability of extensive oncology nursing guidelines and numerous intervention studies, there is still a lack of a clear understanding of the field of oncology nursing practice and whether effective nursing interventions are implemented consistently or credibly evaluated in real-world oncology practice. In oncology nursing practice, no previous research has reported clinical effects without considering context-specific features that affect whether an intervention is adopted, properly implemented among intended patients, and sustained for quality outcome.

Moreover, the measurement tools available for assessing the outcomes of nursing interventions, considering they exist at all, are variable, especially for non-technical care (education, empathy, and communication), and they lack attribution and limit cross-unit learning. As a result, organisations struggle to decide what to scale, where, and under what conditions.

Therefore, this study addresses this gap by applying a realist context–mechanism–outcome (CMO) lens to map the causal pathways through which oncology care contexts provokes mechanisms that shape four implementation outcomes (uptake, consistency, reach, and sustainability).

### 1.1. Middle-Range Theories Supporting the Evaluation of the Impact of Nursing Interventions on Patient Outcomes

Several theories support the evaluation of the impacts of nursing interventions. Donabedian’s theory (2005) comprises a three-component approach, namely, structure, process, and outcomes, and serves as a roadmap to measure the improvements that are used to evaluate the quality of patient care [15]. Determining the barriers to effective nursing interventions from this theoretical perspective will reveal contextual constraints and thus the impact of the interventions provided. Each component within Donabedian’s theory proposes a different measure to evaluate the nursing intervention process:

- The *structure* comprises the input measures that focus on the characteristics of the service or the nurse as a provider and includes the contextual factors that support nursing practice.
- The *process* reflects the operational approach and the work undertaken to deliver the desired outcomes. In nursing practice, it reflects whether the elements of a nursing care plan have been met, with a focus on nursing actions and interventions.
- The *outcome* refers to the impact of applied nursing interventions that reflect whether proper care has been provided for the patient.

Exploring the factors that undermine the impact of nursing interventions on patient outcomes from this theoretical perspective, which focuses on measurement for quality improvement, helps in identifying gaps in the provision of high-quality patient care through the proper application of nursing interventions. Therefore, the interview guide used in this study was inspired by the information from Donabedian’s (2005) middle-range theory [15].

To better align the theoretical paradigm for this study and the generated results, the CMO configuration is used to guide the thematic analysis process by formulating and refining the codes, which clarify how and under what circumstances nursing interventions can achieve high-quality outcomes [16].

### 1.2. Aim

The aim of this study was to explore and analyse the barriers to the effective implementation of nursing interventions in oncology care through a realist lens, delineating the CMO configuration.

## 2. Materials and Methods

### 2.1. Design

This study used a qualitative descriptive approach with semi-structured interviews to elicit nurses’ first-hand accounts of barriers to the implementation of effective nursing interventions in oncology care. This approach is well-suited to producing a rich, practice-proximal description of phenomena without imposing heavy theoretical abstraction and is especially relevant to studying a complex problem when the researcher aims to identify peculiarities and personal opinions [2]. In this case, it enabled a profound comprehension of the participants’ experiences, perceptions, and work-related issues in their practice settings. This study was conducted across oncology units at tertiary hospitals and included a purposive sample of 20 registered nurses involved in the direct care of oncology patients. Interview development and interpretive synthesis were informed by Donabedian’s (2005) [15] middle-range theory. This study followed the Consolidated Criteria for Reporting Qualitative Research (COREQ) guideline [17] to help situate reported barriers across intervention characteristics, characteristics of individuals, and implementation processes. COREQ functioned as a guiding lens rather than a rigid codebook, allowing nurses’ perspectives to remain foregrounded.

### 2.2. Setting

This study was implemented between mid-January 2025 and early May 2025 across three tertiary hospitals where oncology care is provided in inpatient settings. The hospitals were chosen because the diverse healthcare services they offer, their size, and their patient populations enable a general view to be attained of the challenges faced by nurses working in oncology care settings. In two facilities, oncology care is provided in specific oncology units, while in the third facility, it is provided for cancer patients who are admitted to a medical unit where designated beds are reserved for oncology patients. Since these hospitals are governed by the Ministry of Health in Saudi Arabia, they operate based on shared goals and focuses of care. Before commencing this study, the researcher conducted a general assessment to ensure that the settings and practices in these facilities observed the same protocols and policies supporting oncology nursing practice.

### 2.3. Participants

Registered nurses working in oncology settings and providing bedside care were targeted to participate in the current study. This group was chosen because they are direct providers of patient care services and are experienced in providing nursing interventions in oncology care settings. The inclusion criteria were being registered nurses, having at least one year of experience in oncology care, and volunteering to participate. The exclusion criteria were being ‘floating’ nurses in the oncology department, such as students, trainees, or nurses from other departments.

### 2.4. Sampling and Sample Size

A purposeful sampling technique was used to select participants based on their experience of applying bedside nursing interventions in oncology clinical practice [18]. The participants were selected according to three factors: their experience in direct patient care, their engagement in nursing intervention activities, and their willingness to join this study. The allowed range of the final sample size was set to 20 participants, as this number was determined to be sufficient to provide rich, detailed data with a relatively easy scope of analysis. After ethical approval for this study was obtained, the participants were approached by members of the nursing directors’ offices in the tertiary hospitals, who passed on the invitation to participate to registered nurses who met this study’s inclusion criteria.

### 2.5. Data Collection Instruments

The main method of data collection in this study involved semi-structured interviews. The interview guide was created, which included open-ended questions that were designed to capture the obstacles to evaluating nursing interventions. The questions addressed, among other matters, the presence of standardised intervention protocols for oncology practice, resource availability, whether the effect of patient diversity shapes clinical outcomes, and the degree to which different disciplines collaborate in the implementation of nursing interventions. All interviews were conducted by an Assistant Professor in the Faculty of Nursing at Umm Al-Qura University (PhD, Monash University), with formal doctoral training in qualitative methods, substantial experience conducting qualitative studies, and a record of peer-reviewed qualitative publications. The interviewer had no prior relationship with participants and no role in their assessment or supervision. The interviews took place in a private environment to guarantee confidentiality and were recorded with the consent of the interviewees. The interviews lasted 30–60 min each.

The semi-structured interview questions were inspired by Donabedian’s (2005) theory [15]. Open-ended questions to capture the obstacles that hinder the proper application of nursing interventions in oncology nursing practice covered the following areas: the structure, which affects the nurse, as a provider in oncology practice; the process, which focuses on the barriers to providing the essential elements of oncology care based on the nursing intervention care plan; and the outcome, which reflects the impact of nursing interventions on patient outcomes.

Data saturation was defined a priori as (a) no new codes emerging across two consecutive interviews and (b) codebook stability across the next two; empirically, no new codes appeared after interview 18, and the codebook remained stable through interviews 19–20.

### 2.6. Data Analysis

A total of 20 anonymous interview recordings were transcribed verbatim by an independent transcriber who is qualified in dealing with qualitative data, then verified for accuracy by the interviewer. The transcripts were imported into NVivo 12 for analysis. A developed standardised codebook specifying domain-level definitions was used to assign the codes and the emerging themes. From the data, 36 coded references were initially generated. Codes were applied to relevant excerpts and linked to either CMO elements or higher-order themes. The analysis was organised narratively by CMO domain, and analytic memos were maintained throughout to capture emergent concepts and theoretical links. We identified recurring cross-case CMO patterns that articulate plausible causal pathways, that is, how specific contextual conditions in oncology care trigger or constrain mechanisms (e.g., workflow adaptations, professional judgement, team communication) to shape implementation outcomes. Four CMO configurations were ultimately delineated across interviews, and the findings are presented according to these configurations, accompanied by salient quotations from oncology nurses illustrating each CMO.

### 2.7. Ethical Considerations

This study obtained ethical approval from the internal ethical review committee in The Faculty of Nursing in Umm Al-Qura University (UQU) University on 2 January 2025 (Approval No. NSRC-1012025-38). All the participants gave informed consent and were aware of the nature of this study and how their data would be utilised. This study guaranteed the participants’ confidentiality and anonymity, and all identities were removed in the final report. The participants were only interviewed with their consent and could leave this study at any time without consequences.

### 2.8. Trustworthiness

To guarantee reliability in this study, several strategies were adopted to ensure the credibility and validity of the research findings. Credibility was achieved by ensuring there was a long period of engagement: the researcher spent considerable time interacting with the participants to properly understand their experiences. Member checking was employed, wherein the participants were asked to go over the main findings to ensure that they were accurate and in line with their opinions. Transferability was ensured through the provision of multiple descriptions of the setting, participants, and research methods to enable others to evaluate whether the findings apply in other situations with similar contexts. Moreover, direct quotations from the participants were used to increase authenticity by properly representing their views.

## 3. Results

### 3.1. The Demographic Characteristics of the Nurses

This study considered 20 oncology nurses (3 males, 15%; 17 females, 85%). Most held a bachelor’s degree (18, 90%), there were no diploma holders, and two participants (10%) reported having pursued postgraduate studies. The participants had a mean of seven years’ experience. Regarding oncology preparation, 3 nurses (15%) reported a specialised oncology qualification, while 17 (85%) had formal oncology training (Table 1).

### 3.2. Barriers to the Effective Implementation of Nursing Interventions

The analysis yielded four themes, 10 subthemes, and 36 inductive codes (Table 2), centred on ensuring the effective implementation of nursing interventions in oncology clinical practice. The themes captured the pressures of a high-acuity workload and limited real-time evaluation capacity; gaps in technological and measurement infrastructure; staffing and administrative constraints; team-level communication/coordination issues; and patient-level heterogeneity and socioeconomic modifiers. Table 2 summarises each theme with its subthemes, exemplar codes, and CMO alignment. To appraise implementation performance, we applied a four-element checklist of implementation outcomes that comprised uptake, consistency (fidelity), reach, and sustainability across all the subthemes (Table 3). This mapping showed that uptake and consistency were the most constrained by understaffing, administrative burden, and inadequate digital tools; reach was variably limited by fragmented teamwork and patient socioeconomic barriers; and sustainability was threatened by the persistence of high workload without supportive infrastructure.

### 3.3. Barriers to the Effective Implementation of Nursing Interventions in Oncology Clinical Practice

To explore barriers to the effective implementation of nursing interventions in oncology practice, the interview data were analysed using a realist CMO lens (Table 4). The transcripts were coded inductively and synthesised into themes that then were aligned with the C, M, and O elements. CMO configurations were mapped to clarify how oncology-specific contexts (e.g., patient acuity and workload, scarcity of technical support) suppress or trigger mechanisms (e.g., workload adaptation, teamwork, fidelity, patient engagement) to produce outcomes (e.g., uptake, consistency, reach, sustainability). This approach also created context-informed strategies to enhance implementation fidelity and feasibility, thereby strengthening the credible evaluation of effective oncology nursing interventions.

The thematic analysis identified four significant themes that represent the barriers that nurses encounter when assessing the impact of nursing interventions on patient outcomes in oncology facilities.

**Theme 1**.
*Structured safety huddles under high workload.*

**Subtheme 1.1**.
*Patient acuity and workload*.

Limited time was identified as one of the greatest challenges to the assessment of nursing interventions. The environment in which nurses work tends to be action-packed, whereby the turnaround of the patients is fast, and the acuity of the patients is high. One nurse said the following: “*We are always in motion and have no time to sit and keep track of what we are doing for each patient.*” Another reported the following: “*In a critical care situation, we are more concerned with saving lives and caring, but not assessing the outcomes of every action we carry out.*” Such situations allow little space to reflect or even record patient outcomes. The need to immediately assist the patient will override the desire to make an assessment; therefore, nurses may simply use case assessments and not properly collect data through systematic processes. Another barrier to making timely changes to patient care is the inability to assess interventions in real time.

**Subtheme 1.2**.
*Scarcity of technological support*.

The non-existence of technological aids to facilitate the assessment of the nursing interventions was cited by most of the participants. According to one nurse, “*The organisation does not possess the instruments to monitor outcomes as they arise*”; thus, the nurses are restricted to manual processes. The lack of well-developed electronic systems to track patients’ progress or outcomes frustrates the nurses when trying to collect or analyse the data effectively. The lack of such technological resources also makes it harder to trace the effectiveness of the interventions deployed, define the trends, or make wise decisions concerning patient care maintenance.

**Subtheme 1.3**.
*Varying outcome measurements*.

Nurses cannot use standardised tools for measuring nursing interventions to assess outcomes since no such tools yet exist. The main complaint of some participants concerned knowledge of different assessment techniques, which vary across departments or even shifts. One nurse said the following: “*We cannot compare between units how well we are doing when there is no consistent tool we use.*” Another nurse explained the following: “*No common pattern is used to determine whether our interventions are being effective. It complicates monitoring of progress to an extent that it is almost impossible.*” This contradiction confuses what constitutes a successful intervention, as it prevents the noting of longitudinal improvement rates or knowledge-based care changes. Not having a standard strategy is also problematic when it comes to reporting patient outcomes to management and proving the efficiency of nursing interventions to gain the resources needed to move forward with enhancements.

**Subtheme 1.4**.
*Challenges of measuring non-technical interventions*.

The nurses complained about the difficulties of measuring non-technical interventions, including emotional, communicative, and patient education. These are helpful interventions that are subjective and difficult to measure, despite being necessary. One participant said the following: “*How do you quantify anything like empathy or communication?*” “*That is not simple, and we do not have the means to do that.*” Another reported the following: “*You can’t put a number on how comforting a nurse’s words are to a patient. Yet, that’s often the difference in how a patient feels about their care.*” The impossibility of measuring intangible care results in an underestimation of core nursing practice and inhibits the process of assessing the actual contribution of interventions to patient outcomes.

**Theme 2**.
*Protocol without backup staff.*

**Subtheme 2.1**.
*Understaffing*.

The shortage of resources, specifically staff, was reported to be a significant barrier to the assessment of nursing interventions. Most nurses are working in short-staffed conditions, which leads to huge workloads and little time to evaluate patient outcomes. One of the nurses said the following: “*We are usually understaffed, and when that becomes the case, the attention switches from evaluation to mere survival of the shift.*” According to another nurse, it is hard to pay close attention to patients and assess the results of care when there are not enough staff. This staff shortage complicates the tasks of the nurses and prevents them from spending enough time with patients, gathering data, or tracking interventions. Moreover, the need to accomplish more tasks with fewer resources renders it even more challenging to conduct a systematic analysis of patient outcomes and find gaps in care practice.

**Subtheme 2.2**.
*Administrative burden*.

Patient care is accompanied by administrative activities, which further increase time constraints. The issue that the nurses were the most concerned about was the time spent completing electronic health record (EHR) documentation and their administrative tasks. One nurse related that she often finds herself without the energy and time to evaluate her interventions after completing all paperwork. A fellow nurse said the following: “*It is paperwork without end. We are supposed to write anything down, and yet we have no time left to follow up on the patient’s progress in any meaningful manner.*” This administrative load undermines nurses’ capability to concentrate on the proper assessment of their actions and reduces the number of opportunities to give direct patient care.

**Theme 3**.
*Ineffective intradisciplinary team collaboration.*

**Subtheme 3.1**.
*Inefficient communication in healthcare teams*.

Another major obstacle to the assessment of nursing interventions was the lack of relationships and cooperation between healthcare teams. The nurses frequently complained about their inability to work collaboratively with the rest of the healthcare team: “*Sometimes, we could not tell what the doctors are up to, let alone how our care fits into theirs.*” Another added the following: “*Due to communication failures, fractured care is obtained. We do not always get the whole picture of what goes on with the patient.*” This communication failure introduces some gaps in patient care, which makes it challenging to review the overall effect of nursing interventions. The lack of interdisciplinary cooperation implies that the nurses might not be able to access all the patient data and outcomes, which hinders their understanding of the real effect of their services on patients.

**Subtheme 3.2**.
*Disintegrated patient care*.

Another problem associated with care that might be caused by poor teamwork is the fragmentation of patient care. The nurses mentioned that even when complete and consistent information is gathered, it cannot always be communicated by other professionals, and patient outcomes cannot, therefore, be assessed properly: “*We’re supposed to be working together, but when information isn’t shared, it’s like we’re all working in silos. The alignment of the team is necessary when it is difficult to measure the results of care.*” This disintegration in the care delivery process does not allow for performing rigorous assessments and minimises the chances of nursing interventions being properly evaluated within the context of total patient care.

**Theme 4**.
*Patient heterogeneity and contextual modifiers.*

**Subtheme 4.1**.
*Complex health needs and comorbidities*.

It is not easy to attribute changes in health outcomes in the acute care setting to nursing interventions because of the complexity of and variability in patients. Nurses often work with patients with several health conditions; thus, it is not easy to identify the effects of nursing care. One of the nurses said the following: “*Every patient is unique, and he or she brings his or her problems.*” According to another nurse, “*Patients do not have one condition; they can have several diagnoses, and every single medical condition can complicate the process of recovery.*” This inconsistency makes the scoring process more cumbersome because the evaluating nurses have to consider many factors that affect the outcomes of patients that can vary significantly, such as comorbidities, drugs, or patient reactions.

**Subtheme 4.2**.
*Patient preferences and socioeconomic factors*.

Patient preferences and other socioeconomic factors also hinder the evaluation of nursing interventions. One nurse encountered patients who had particular wishes concerning how they should be treated, and quantifying success in cases where patients did not adhere to the prescribed interventions was difficult. According to one nurse, “*Patients with lower socioeconomic statuses sometimes have reduced access to follow-up treatment; then, it becomes challenging to determine the success of the nursing intervention in conditions of long-term assessment.*” Another complexity in outcome evaluation results from the variability in patient engagement and compliance with care plans. Moreover, it may be difficult to determine the overall effect of nursing care due to the socioeconomic status of patients and the resources available to them, which may affect their responsiveness to treatment.

## 4. Discussion

Guided by a realist (CMO) lens, the study findings explain *how* oncology care contexts activate (or suppress) mechanisms that shape four implementation outcomes: uptake, consistency (fidelity), reach, and sustainability (Table 3). Four themes were identified, (1) structured safety huddles under conditions of high workload; (2) protocols without backup staff; (3) ineffective intradisciplinary team collaboration; and (4) patient heterogeneity and contextual modifiers, with ten subthemes (Table 2).

These findings indicate that it is challenging to credibly evaluate the effectiveness of nursing interventions, not because they possess no intrinsic merit but because contexts and mechanisms obscure their observable effects.

The participants consistently described having “no time to assess”, high acuity/rapid turnover, life-saving tasks taking priority over evaluation, missed/late documentation, minimal reflection time, and no real-time assessment capacity. These pressures were amplified by a lack of technology support: “no tools to monitor outcomes,” the burden of manual tracking, EHRs that were not fit for the purpose, data fragmentation, and the absence of outcome dashboards. In CMO terms, this context of relentless demand and a weak information technology system triggers triage and workaround mechanisms, which can adversely impact uptake, consistency, and sustainability. This finding aligns with previous studies that revealed that inconsistent measures and unhelpful digital systems push clinicians towards subjective judgements and selective documentation, undermining reliability and longitudinal comparability [19,20]. Studies have therefore called for streamlined, standardised outcome sets and point-of-care prompts [8].

Protocols without backup staff or with chronic short-staffing, workload overload, and heavy administrative burden (“paperwork without end”) shift nurses’ attention from evaluation to ‘surviving the shift’, limiting patient contact time and the ability to track interventions systematically.

These contexts simulate coping mechanisms (task compression, deprioritisation of evaluation), depressing uptake and consistency, constrictive reach across shifts and units, and weakening sustainability due to fatigue and drift. This pattern resonates with evidence linking staffing deficits to quality shortfalls and with research showing that documentation burden leaves little time for reflective practice [21,22,23]. Where fit-for-purpose health IT has been implemented, the completeness and timeliness of evaluation improve, supporting the recommendation that IT should be more effectively deployed such as in the use of auto-pull signals, outcome dashboards, and single-click fidelity checks [24,25].

Ineffective intradisciplinary collaboration, poor nurse–physician information flow, lack of shared plans of care, fractured information leading to fractured care, incomplete access to outcome data, siloed working, inconsistent handovers, and misaligned goals decouple nursing actions from team outcomes. Here, the context of weak coordination causes those in each profession to work only towards their own targets, yielding incomplete datasets and ambiguous attribution. The result is uneven consistency and reach, with consequent risks for sustainability. These observations show that structured interprofessional routines and shared visibility (concise plans of care, evaluation checkpoints) enhance fidelity and coverage in complex service [26,27,28].

Finally, patient heterogenicity and the contextual modifiers of high case-mix complexity, multiple diagnosis, polypharmacy/toxicity, variable reactions, preference-driven non-adherence, limited follow-up, variable engagement, and socioeconomic constraints blur the signals of effects and complicate attribution. These contexts activate adaptation mechanisms (case-by-case tailoring) that, if safeguards are not in place, erode consistency and restrict reach to more advantaged groups. Sustainability suffers when successful adaptations are neither captured nor spread. Previous work similarly shows that socioeconomic position and health literacy shape adherence and outcomes, while routine patient-reported outcomes (PROs) can improve the effective implementation of nursing interventions despite heterogeneity [29,30].

Across the themes identified, CMO mapping clarifies where change yields results most quickly. Uptake is the most constrained by understaffing, time pressure, and missing digital supports; consistency suffers when measures vary, paperwork creates load, and team routines are weak; reach is restricted by fragmented teamwork and SES barriers; sustainability is harmed by chronic workload and the absence of supportive infrastructure. The following targeted action mechanisms are recommended: (1) time for staff to carry out evaluations should be protected via brief, predictable micro safety huddles with the authority to require interventions; (2) minimal standardisation should be introduced by drawing up a core set of outcomes (including a brief PRO) and one or two point-of-care fidelity checks; (3) IT should be deployed more effectively (auto-pull vitals/labs, outcome dashboards, single-click prompts); (4) enough staff should be made available to allow for evaluation by recognising evaluation time in the rostering; (5) the visibility of patient care across teams should be enabled by using a one-page shared plan of care and handover evaluation checkpoints; and (6) structures should be adapted with appropriate rules, and adaptations should be routinely recorded.

The findings of this study, which align with the broader literature, suggest that the primary threats to the effective implementation of nursing interventions in oncology care can be resolved. Aligning staffing models, measurement, digital tools, and team routines with the mechanism identified in this study can improve uptake, consistency reach, and sustainability, thereby strengthening both the implementation and evaluability of nursing interventions in oncology care.

## 5. Conclusions

The results of the present study are important in understanding the barriers hindering the process of measuring the effectiveness of nursing interventions on patient outcomes in an oncology care environment among nurses. Given that this study was conducted in general tertiary hospitals rather than a dedicated oncology centre, the interview findings are best framed as perceived context-specific barriers within one complex with plausible implications for the care process. The lack of uniform instruments of assessment, time pressure, limited resources, individual differences in patients, and the inability to collaborate across disciplines are all factors that challenge nurses in terms of efficiently measuring their actions. These obstacles impair the effective assessment of patient outcomes by nurses and restrict their ability to improve their practice according to evidence. Such barriers considerably affect the quality of patient care in acute settings; thus, attention should be paid to dismantling these obstacles to enhance nursing practice and patient outcomes. This study is limited by a small sample which constrains transferability; additionally, as qualitative analysis is interpretive, the findings may be influenced by potential researcher bias despite the rigorous methodology used.

## Figures and Tables

**Table 1:** Demographic characteristics of participating nurses (N = 20).

Characteristic	N (%), Mean
**Gender**	
Male	3 (15%)
Female	17 (85%)
**Education level**	
Diploma	0
Bachelor	18 (90%)
Postgraduate studies	2 (10%)
**Years of experience**	Mean = 7 years
**Oncology speciality**	
Specialised qualification	3 (15%)
Formal training	17 (85%)

**Table 2:** Barriers identified by nurses.

Themes	Subthemes	Codes
Theme 1: Structured safety huddles under high workload	1.1 Patient acuity and workload	“No time to assess” High acuity/rapid turnover Life-saving tasks > evaluation Missed/late documentation Minimal reflection time No real-time assessment capacity
	1.2 Scarcity of technological support	“No tools to monitor outcomes” Manual tracking burden Electronic health records (EHRs) not fit for purpose Data fragmentation No outcome dashboards/analytics
Theme 2: Protocol without backup staff	2.1 Understaffing	Chronic short staffing Workload overload Evaluation deprioritised (“survive the shift”) Limited patient contact time Inability to track interventions systematically
	2.2 Administrative burden	EHR paperwork overload Time/energy depletion after admin “Write everything” culture Reduced direct care opportunity
Theme 3: Ineffective intradisciplinary team collaboration	3.1 Inefficient communication	Poor nurse–physician information flow Lack of shared plans of care Fractured information → fractured care Incomplete access to outcome data
	3.2 Disintegrated patient care	Siloed working Inconsistent handover/information sharing Misaligned team goals Evaluation not embedded in team process
Theme 4: Patient heterogeneity and contextual modifiers	4.1 Complex needs and comorbidities	High case-mix complexity Multiple diagnoses confound attribution Polypharmacy/toxicity effects Variable patient reactions
	4.2 Preferences and socioeconomic factors	Preference-driven non-adherence Limited follow-up access (SES) Variable engagement with care plan Resource constraints shape responsiveness

**Table 3:** Outcome focus implementation from the CMO configuration for four implementation outcomes (✓ = strong effect; (•) = moderate/indirect).

Theme/Subtheme	Uptake (Adoption)	Consistency (Fidelity)	Reach (Coverage)	Sustainability (Over Time)
1.1 Patient acuity and workload	✓	✓	(•)	✓
1.2 Scarcity of technological support	✓	✓	✓	✓
2.1 Understaffing	✓	✓	✓	✓
2.2 Administrative burden	(•)	✓	(•)	✓
3.1 Inefficient communication	(•)	✓	✓	(•)
3.2 Disintegrated patient care	(•)	✓	✓	(•)
4.1 Complex needs and comorbidities	(•)	✓	(•)	(•)
4.2 Preferences and socioeconomic factors	(•)	(•)	✓	(•)

**Table 4:** CMO configuration of barriers to effective implementation of nursing interventions in oncology clinical practice.

**Theme 1: Structured safety huddles under high workload**	**CMO-1: Structured safety huddles under high workload** **Results:** Effective implementation of nursing interventions and quality outcomes. Nurses work with multiple patients in the oncology unit with acuity that varies from low to high, depending on the severity of conditions, under significant time pressure, and they must handle therapeutic protocols and a varying complexity of symptoms. The complexity of oncology patient care leads to a heavy workload, leaving little opportunity to document and evaluate the applied interventions (*context*). This requires coordination via shared priorities that categorise patients based on their acuity level, available through standardised protocols for oncology care and point-of-care documentation prompts (*mechanism*). Attentive and customised care should be provided for cancer patients, as the chances of deterioration and escalation are minimised according to the care provided (*outcome*). **Interview scripts:** ***Subtheme 1.1: Patient acuity and workload*** Limited time was identified as one of the greatest challenges to the assessment of nursing interventions. The environment in which nurses work tends to be action-packed, whereby the turnaround of the patients is fast, and the acuity of the patients is high. One nurse said the following: “*We are always in motion and have no time to sit and keep track on what we are doing for each patient.*” Another reported the following: “*In a critical care situation, we are more concerned with saving lives and caring, but not assessing the outcomes of every action we carry out.*” Such circumstances allow little space to reflect or even record patient outcomes. The need to immediately assist the patient will override the desire to make an assessment; therefore, nurses may simply use case assessments and not properly collect data through systematic processes. Another barrier that does not allow for making timely changes to patient care is the inability to assess interventions in real time. Link to CMO: This subtheme specifies the *context* that necessitates safety huddles; the huddles operationalise shared priorities so that outcome assessments are not lost in the rush. ***Subtheme 1.2: Scarcity of technological support*** The non-existence of technological aids to facilitate the assessment of the nursing interventions was cited by most of the participants. According to one nurse, “*The organisation does not possess the instruments to monitor outcomes as they arise*”; thus, the nurses are restricted to manual processes. The lack of well-developed electronic systems to track the progress of patients or outcomes frustrates the nurses when trying to collect or analyse data effectively. The lack of such resources makes it harder to trace the effectiveness of the interventions deployed, define the trends, or make wise decisions concerning patient care maintenance. Link to CMO: This explains why the *mechanism* includes point-of-care documentation prompts: safety huddles coupled with simple, immediate HER prompts (or paper micro-checklists) compensate for limited technological resources, maintaining the data flow needed for timely evaluation and escalation. ***Subtheme 1.3: Varying outcome measurements*** Nurses cannot assess outcomes without adhering to standardised tools for measuring nursing interventions since no such tools yet exist. The main complaint of some participants concerned knowledge of different assessment techniques, which vary across departments and even shifts. One nurse said the following: “*We cannot compare between units how well we are doing when there is no consistent tool we use.*” Another nurse explained the following: “*No common pattern is used to determine whether our interventions are being effective. It complicates monitoring of progress to an extent that it is almost impossible.*” This contradiction confuses what constitutes a successful intervention, which prevents the possibility of noting longitudinal improvement rates or knowledge-based care changes. Not having a standard strategy is also problematic when it comes to reporting patient outcomes to management and proving the efficiency of nursing interventions to gain the resources needed to move forward with enhancements. Link to CMO: This subtheme explains that when the *context* involves unit-to-unit variability and the absence of standardised assessment tools, the *outcome* is that nurses cannot compare performance or define improvements. ***Subtheme 1.4: Challenges of measuring non-technical interventions*** The nurses complained about the difficulties of measuring non-technical interventions, including emotional, communicative, and patient education. These are helpful interventions that are subjective and difficult to measure, despite being necessary. One nurse said the following: “*How do you quantify anything like empathy or communication? That is not simple, and we do not have the means to do that.*” Another reported the following: “*You can’t put a number on how comforting a nurse’s words are to a patient. Yet, that’s often the difference in how a patient feels about their care.*” This impossibility of measuring intangible care results in an underestimation of core nursing practice and inhibits the process of assessing the actual contribution of interventions to patient outcomes. Link to CMO: This explains that when the *context* involves non-technical care that lacks standardised, quantifiable measures, the *mechanism* is that subjectivity and the absence of agreed metrics contribute to patient *outcomes* that cannot be credibility assessed.
**Theme 2: Protocol without backup staff**	**CMO-2: Protocol without backup staff** **Results:** Compliance without reasoning ‘box-ticking’, improvement in vanish off-shifts, and silent escalation failure. Understaffed shifts, multiple competing alarms, and missed care from the nursing care plan were noted (*context*). Workarounds and the deprioritisation or delaying of some simple elements from the nursing care plan, such as basic care nursing interventions, and providing incomplete care protocol were mentioned (*mechanism*). The participants also spoke of trigger-to-action delays and the chance of deterioration and ICU transfers (*outcome*). **Interview scripts:** ***Subtheme 2.1: Understaffing*** The shortage of resources, specifically staffing, was reported as a significant barrier to the assessment of nursing interventions. Most nurses are working in short-staffed conditions, which leads to huge workloads and little time to evaluate patient outcomes. One nurse said the following: “*We are usually understaffed, and when that becomes the case, the attention switches from evaluation to mere survival of the shift.*” According to another nurse, it is hard to pay close attention to the patients and assess the results of care when there are not enough staff members. This staff shortage complicates the tasks of the nurses and prevents them from spending enough time with patients, gathering data, or tracking interventions. Moreover, the need to accomplish more tasks with fewer resources makes it more difficult to conduct a systematic analysis of patient outcomes and identify the gaps in care practice. Link to CMO: In this resource-constrained context, the pressure and alarm burden precipitate workarounds (*mechanism*), which, in turn, postpone escalation and suppress outcome evaluation, creating the observed deterioration and transfers (*outcome*). ***Subtheme 2.2: Administrative burden*** Patient care is accompanied by administrative activities, which further increase the time constraint. The issue that nurses were the most concerned about was the time spent completing EHR documentation and their administrative tasks. One nurse often finds herself without the energy and time to evaluate her interventions after completing all paperwork. A fellow nurse said the following: “*It is paperwork without end. We are supposed to write anything down, and yet we have no time left to follow up on the patient’s progress in any meaningful manner.*” This administrative load undermines nurses’ capability to concentrate on the proper assessment of their actions and reduces the number of opportunities to give direct patient care. Link to CMO: When the *context* includes a heavy administrative workload, the time scarcity and cognitive overload that divert attention from evaluation (*the mechanism*) lead to *outcomes* where assessments of interventions are deprioritised.
**Theme 3: Ineffective intradisciplinary team collaboration**	**CMO-3: Ineffective intradisciplinary team collaboration** **Results:** Order conflicts, rise in patient risk, unclear roles, ineffective communication. Parallel workstreams among the healthcare providers without a shared plan or clear lead were noted (*context*). The ambiguity of the provided care, diffusion of responsibility, and hierarchy-driven silence were mentioned. Delayed interventions, and thus the misaligned application of nursing interventions, increase the chances of missed care (*outcome*). ***Subtheme 3.1: Inefficient communication in healthcare teams*** Another major obstacle to the assessment of nursing interventions was the non-existent relationship and cooperation between healthcare teams. Several nurses complained about their inability to work collaboratively with the rest of the healthcare team. One nurse said the following: “*At times, we could not tell what the doctors are up to, let alone how our care fits into theirs.*” Another added the following: “*Due to the communication failure, care is fractured. We do not always get the whole picture of what goes on with the patient.*” This communication failure introduces gaps into patient care, which makes it challenging to review the overall effect of nursing interventions. A lack of interdisciplinary cooperation implies that nurses might not be able to access all the patient data and outcomes, which hinders their understanding of the real effect of their services on patients. Link to CMO: This specifies the *context* (poor cross-team coordination) that drives the *mechanism* (ambiguity/silence), yielding the *outcome* (misaligned, delayed interventions and missed care). ***Subtheme 3.2: Disintegrated patient care*** Another problem associated with care that might be caused by poor teamwork is the fragmentation of patient care. The nurses mentioned that complete and consistent information is sometimes gathered but cannot always be communicated by other professionals; therefore, patient outcomes cannot be assessed properly. One nurse said the following: “*We’re supposed to be working together, but when information isn’t shared, it’s like we’re all working in silos. The alignment of the team is necessary when it is difficult to measure the results of care.*” This disintegration in the care delivery process does not allow for performing rigorous assessments and minimises the chances of nursing interventions being properly evaluated within the context of total patient care. Link to CMO: This illustrates that missing shared plans and real-time updates (*context*) induce the diffusion of responsibility (*mechanism*), leading to delayed, misaligned care and missed opportunities to evaluate and escalate (*outcome*).
**Theme 4. Patient heterogeneity and contextual modifiers**	**CMO-4: Patient heterogeneity and contextual modifiers** **Results:** Greater difficulty attributing outcomes to specific nursing interventions, inconsistent effect sizes across cases, heavier scoring burden, and delayed/fragmented evaluation were found. Oncology units have patient heterogeneity: multimorbidity, polypharmacy, fluctuating, trajectories, diverse preferences, and socioeconomic constraints (access, follow-up, engagement) (*context*). Protocols require individualised deviation and prioritisation; adherence varies; competing conditions and patient choices introduce ‘signal noise’, creating attribution ambiguity and reducing the comparability of outcome data (*mechanism*). It becomes more difficult to link interventions to outcomes in real time; there are inconsistent metrics across patients, leading to the missed or delayed recognition of intervention impact and reduced confidence in evaluations (*outcome*). ***Subtheme 4.1: Complex health needs and comorbidities*** It is not always easy to attribute changes in health outcomes in the acute care setting to nursing interventions because of the complexity of and variability in patients. Because nurses often work with patients with several health conditions, it is not easy to identify the effects of nursing care. One nurse said the following: “*Every patient is unique, and he or she brings his or her problems.*” According to another, “*Patients do not have one condition; they can have several diagnoses, and every single medical condition can complicate the process of recovery.*” This inconsistency makes the scoring process more cumbersome because the evaluating nurses have to consider many factors that affect the outcomes of patients that can vary significantly, such as comorbidities, drugs, or patient reactions. Link to CMO: This subtheme explains that when the *context* is complex, *the mechanism* attributes uncertainty, and confounders lead to unreliable comparisons, and nursing care and scoring become cumbersome (*outcome*). ***Subtheme 4.2: Patient preferences and socioeconomic factors*** Nursing interventions cannot be evaluated easily due to patient preferences and other socioeconomic factors. One nurse encountered patients who had their own wishes concerning how they should be treated, and quantifying success in cases where patients did not adhere to the prescribed interventions is hard. According to one nurse, “*Patients with lower socioeconomic statuses sometimes have reduced access to follow-up treatment; then, it becomes challenging to determine the success of the nursing intervention in conditions of long-term assessment.*” Another complexity of outcome evaluation is a result of the variability in patient engagement and compliance with plans of care. Moreover, it may be difficult to determine the overall effect of nursing care due to the socioeconomic status of patients and the resources available to them, which may affect their responsiveness to treatment. Link to CMO: Evaluation is further complicated by preference-sensitive choices and resources constraints. Some patients decline or modify elements of care; other face barriers to follow-up. Lower socioeconomic status can limit access and adherence, obscuring long-term *outcome* attribution.

italics indicates elements of the Context-Mechanism-Outcome (CMO) Configuration.

## Data Availability

The data presented in this study are available on request from the corresponding author due to participant privacy and ethical restrictions approved by the Institutional Review Board.
